# Cost of Type 2 Diabetes Patients with Chronic Kidney Disease Based on Real-World Data: An Observational Population-Based Study in Spain

**DOI:** 10.3390/ijerph18189853

**Published:** 2021-09-18

**Authors:** Ruth Usó-Talamantes, Silvia González-de-Julián, Javier Díaz-Carnicero, Inmaculada Saurí-Ferrer, José Luis Trillo-Mata, Marc Carrasco-Pérez, Jorge Navarro-Pérez, José Luis Górriz, David Vivas-Consuelo, Josep Redón

**Affiliations:** 1 Conselleria de Sanitat i Salut Pública, Generalitat Valenciana, 46010 Valencia, Spain; 2 School of Medicine and Health Sciences, Valencia Catholic University, 46001 Valencia, Spain; 3 Research Unit for Health Economics and Management, Universitat Politècnica de València, 46022 Valencia, Spain; silgonde@upv.es (S.G.-d.-J.); jadiacar@upv.es (J.D.-C.); dvivas@upv.es (D.V.-C.); 4 INCLIVA Research Institute, 46010 Valencia, Spain; isauri@incliva.es (I.S.-F.); navarro_jorge@gva.es (J.N.-P.); jlgorriz@gmail.com (J.L.G.); josep.redon@uv.es (J.R.); 5 Hospital Valencia Clínico–Malvarrosa, 46010 Valencia, Spain; trillo.jlu@outlook.com; 6 Boehringer Ingelheim España S.A., 08174 Barcelona, Spain; marc.carrasco@boehringer-ingelheim.com; 7 School of Medicine, University of Valencia, 46010 Valencia, Spain

**Keywords:** chronic kidney disease, type 2 diabetes, costs, healthcare resources, burden of care, KDIGO classification, Clinical Risk Groups

## Abstract

This study analyzed the prevalence, costs and economic impact of chronic kidney disease CKD in patients with T2D in a Spanish Health District using real-world data. Observational cross-sectional study in adult patients with T2D was through data extracted from the information systems of the Valencia Clínico–La Malvarrosa Health District in the year 2015. Patients were stratified with the KDIGO classification for CKD. Additionally, patients were assigned to Clinical Risk Groups (CRGs) according to multimorbidity. Direct costs of primary and specialized care, and medication were estimated. The prevalence of T2D in the database population (*n* = 28,345) was 10.8% (mean age (SD) = 67.8 years (13.9); 51.5% male). Up to 14.935 patients (52.6%) had data on kidney function. According to the KDIGO classification, 66.2% of the patients were at low risk of CKD, 20.6% at moderately increased risk, 7.9% at high risk, and 5.2% at very high risk. The average healthcare costs associated with these four risk groups were EUR 3437, EUR 4936, EUR 5899 and EUR 7389, respectively. The large number of T2D patients with CKD in the early stages of the disease generated a significant increase in direct healthcare costs. The economic impact could be mitigated by early and comprehensive therapeutic approaches.

## 1. Introduction

In 2019, approximately 463 million adults (20–79 years) had diabetes worldwide [1]. By 2030, around 552 million people in the world will suffer from diabetes (700 million by 2045), the global prevalence will rise to 9.9% and the economic and human costs will be huge [2]. In Spain, the overall prevalence of diabetes (adjusted for age and sex) was 13.8% in 2010 (95% CI 12.8, 14.7%), of which about half had unknown diabetes (6.0%, 95% CI 5.4, 6.7%), but up to 30% of the population had some impairment in carbohydrate metabolism [3]. Type 2 diabetes (T2D) is the most common type of diabetes, accounting for around 90% of all diabetes cases [1]. The prevalence of T2D increases with age [4] and is higher in men than in women up to 74 years of age, after which this relationship is reversed [5].

Patients with T2D have an increased risk for macro- and microvascular diseases such as coronary heart disease, peripheral arterial disease, cerebrovascular accident, retinopathy, chronic kidney disease (CKD) and end-stage renal disease. CKD is present in about one third of patients with T2D, and diabetes is considered the most common cause of end-stage renal disease. The prevalence of CKD associated to T2D (estimated at 26–27%) has stabilized in most developed countries in the last decade, but is increasing in some population groups [6]. In Spain, the prevalence of CKD among patients with T2D ranged from 27.9 to 34.1 [7,8,9], being 37.2% among patients aged ≥ 65 years [10]. Given that ageing and obesity are implicated in T2D and CKD and both are becoming increasingly common, the socioeconomic impact of this comorbid disease is huge [11]. In patients with T2D, CKD is associated with increased total and cardiovascular mortality [12,13], and it can also potentially lead to kidney failure requiring dialysis or a kidney transplant. In Spain, diabetic kidney disease presents a progressively increasing incidence and it is the first cause of initiation of renal replacement therapy, reaching 36.7% in the 2019 National Renal Patients Registry [14].

The direct costs of diabetes are estimated to represent 8% of the total Spanish National Health System (NHS) expenditures [15,16]. Medication and hospitalization are the main drivers of costs [16,17]. The main factor associated to the high cost of management of patients with T2D is the treatment of complications, which account for 71.4% of the total direct costs of T2D [18]. However, although there are some studies on costs of T2D and its complications at the regional level [19,20,21,22,23,24,25], there is limited evidence of the socioeconomic impact and burden of CKD in patients with T2D in the public health system. Additionally, several studies have shown that healthcare costs dramatically increase with deteriorating renal function, especially in the later stages of the disease, highlighting the need of identifying and mitigating factors that promote CKD progression [26,27,28,29]. Although the high costs associated with end-stage kidney disease (e.g., kidney transplantation, hemodialysis) are known, it would be of interest to determine the associated costs based on CKD risk stages, and to identify the key factors that contribute to cost increments in Spain in those patients. The objective of this study was to evaluate the use of healthcare resources and the total costs per patient in patients with T2D and CKD, using real-world database information from a health department in Spain.

## 2. Materials and Methods

### 2.1. Study Design

This was an observational, population-based, cross-sectional study on the use of resources and associated costs with CKD in patients with T2D as a function of CKD stage and comorbidities in the period from 1 January to 31 December 2015.

Risk of CKD was estimated using the KDIGO (Kidney Disease: Improving Global Outcomes) 2012 criteria, which classifies the different stages of kidney disease based on estimated glomerular filtration rate (eGFR) and albumin excretion [30]. The closest available values of these parameters to 31 December 2015 were used to classify each patient in a KDIGO group.

To further characterize the patients, they were also classified in Clinical Risk Groups (CRGs) according to the main complications and comorbidities related to T2D [31]. CRGs have been used before in this population group to estimate burden of care and T2D comorbidity [20,32]. CRGs assign patients to mutually exclusive categories and a severity level according to their chronic health condition: (1) healthy; (2) significant acute disease; (3) single minor chronic disease; (4) minor chronic disease in multiple organ systems; (5) single moderate dominant or chronic disease; (6) significant chronic disease in multiple organ systems; (7) dominant chronic disease in three or more organ systems; (8) dominant neoplasms, metastases and complications, and 9) severe diseases or extreme healthcare needs.

### 2.2. Data Sources

Data for the study were obtained from the electronic outpatient clinical records (ABUCASIS) that include the Ambulatory Information System (SIA) and the Pharmacy Prescriptions Manager (GAIA); the Hospital Minimum Data Set (MDS), the Population Information System (SIP), the Economic Information System (SIE), the database for emergency services from the Hospital Information System (HIS) and the databases from the Hospital Pharmacy of the Regional Health Department. The clinical analytical laboratory information have been obtained from Geslab.

Patient data were anonymized and introduced in a purpose-built database in which each record corresponded to a single patient. The study has strictly complied with the current personal data protection regulations, specifically Regulation 2016/679 of the European Parliament and of the Council of 27 April 2016 on the protection of natural persons with regard to the processing of personal data and on the free movement of such data, as well as the Organic Law 3/2018, of December 5, concerning protection of personal data and guarantee of digital rights.

Additionally, the study protocol was approved by the Ethical Review Board of the University Hospital Clínico of Valencia.

### 2.3. Study Population

Patients aged ≥ 18 years registered in the Population Information System (SIP) of the Valencian Community corresponding to the Valencia Clínico–La Malvarrosa Health District were identified from the database comprising all patients from the Valencian Community. The Health Department’s population in 2015 was 320,956. The inclusion criteria were the following: (a) patients with International Classification of Diseases-9th Revision- Clinical Modification (ICD-9-CM) codes 250 (diabetes mellitus) and subcategories; codes related to the use of test strips (648.8, 648.0, 775.1, 790.6, 790.29, 707.10, 707.9, 731.8, 251.2, 211.7), and code 249 (secondary diabetes) and its subcategories; (b) patients with prescribed medication with antidiabetic active ingredients according to the Anatomical Therapeutic Chemical Classification System (ATC) in groups A10 (antidiabetics) and V04CA (blood glucose test strips), and (c) patients with Major Diagnostic Category (MDC) 101 (diabetes) and/or Episode Disease Category (EDC) 424 (diabetes).

### 2.4. Cost Analysis

Resource consumption related to clinical results, hospitalization costs and pharmaceutical expenses were calculated for each patient, as well as the average cost for each stage of kidney disease. Total annual costs were computed by addition of primary and specialized care, and pharmaceutical costs in the reference year 2015. For primary care (primary care physician and nursing), outpatient consultations, and kidney transplants, the standard unit costs were derived from the Tax Law of the Generalitat Valenciana of 2015. Laboratory costs for each specific test were provided by the Clinic Hospital of Valencia. Imaging costs were calculated taking as relative unit costs (RUC) a thorax X-ray (EUR 25.31). Dialysis costs were established taking as reference a mean cost of hemodialysis of EUR 23,049 patient/year and of peritoneal dialysis of EUR 15,487 per patient/year [33]. The cost assigned to patients at dialysis for less than a year was proportional to the number of days and type of dialysis.

### 2.5. Statistical Analysis

Descriptive statistics using frequencies, percentages and cross tabulations were used.

Based on the costs per patient obtained, these costs were analyzed by KDIGO groups. First, the distribution was analyzed by calculating confidence intervals for each category of the KDIGO scale. From this, differences between cost distributions by risk group were tested using the two-sample Kolmogorov–Smirnov test. In this test, the null hypothesis, that the data from the two distributions to be compared come from the same continuous distribution, is tested.

A multiple linear regression model was used to evaluate the determinants of the total cost. For the selection of variables to be introduced into the model, results from eGFR and urinary albumin to creatinine ratio were first considered. However, as the explanatory level with these two variables was insufficient, ACRG3 categories of health status and severity were also introduced as variables. Finally, an additive selection of variables was used in the regression, selecting only those statistically significant, and that increased the explanatory power of the model.

## 3. Results

### 3.1. Population Characteristics

The total population of the Valencia Clínico–La Malvarrosa Health District was 263.334 adults in 2015. Of these, 28,345 patients with T2D were identified in the database. The mean (SD) age was 67.8 (13.9) years and 51.5% were male. The prevalence of T2D was 10.8%, slightly higher in men (11.7%) than in women (9.9%). The age groups with highest prevalence were those in the range of 56–70 years (35.1%) and in the range of 71–85 years (38.8%). Glycated hemoglobin (HbA1c) was controlled (≤7%) in 58.5% of patients aged <65 years, while in patients ≥ 65 years, the HbA1c was controlled (≤8%) in 86.1%.

From the total population, a subgroup of 14,935 patients with T2D and data on eGFR and albuminuria were considered for further analysis. In these patients, the mean (SD) age was 68.3 (13.0) years and 52.8% were male. Table 1 shows the distribution of these patients according to CRG. Most patients (87.4%) could be classified in CRGs 5 (26.7%), 6 (56.5%) or 7 (11.8%). Table 2 shows the same patients classified according to the KDIGO 2012 criteria [30]. According to eGFR, the most frequent category (44.9% of the patients) was G2 (60–89 mL/min/1.73 m^2^) and according to albuminuria, the vast majority (78.7%) were in the A1 category (<30 mg/g). Overall, 66.2% of the patients were classified as ‘low risk’, 20.6% as ‘moderately increased risk’, 7.9% as ‘high risk’, and 5.2% as ‘very high risk’, indicating that 33.8% of the patients in this population were at risk of CKD.

### 3.2. Use of Healthcare Resources

The use of healthcare resources increased proportionally to the patients’ KDIGO categories (from low to the very highly increased risk) for all types of resources (Table 3). Primary care and outpatient consultations were the most common resources used by the patients, with a mean of 19.32 and 4.21 visits per patient, respectively.

The costs of each resource also increased with risk categories. Hospital admissions and pharmacy expenditure were the highest types of costs in these patients (EUR 1197 and EUR 1308, respectively).

### 3.3. Costs in Each KDIGO Category and Distribution of Costs

**Figure 1:**
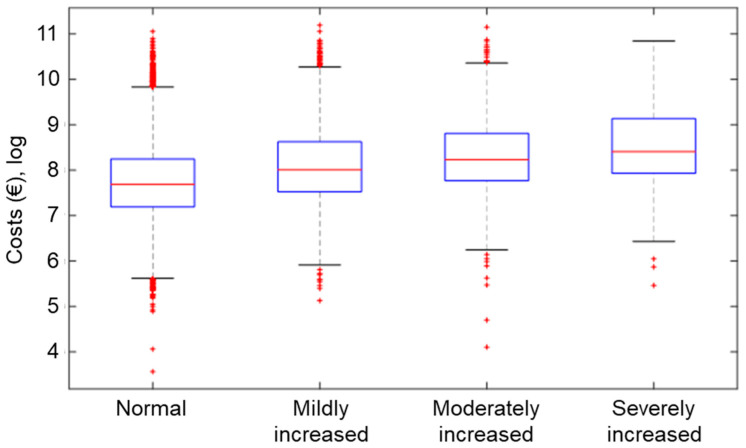
Box plots of the total mean costs (log scale) associated to KDIGO risk groups.

Mean overall costs associated to each risk category increased greatly as eGFR decreased and albuminuria increased (Table 4). The mean (95% CI) costs per patient were EUR 4149 (4066–4231). Costs increased with each category of risk (Figure 1).

**Figure 2:**
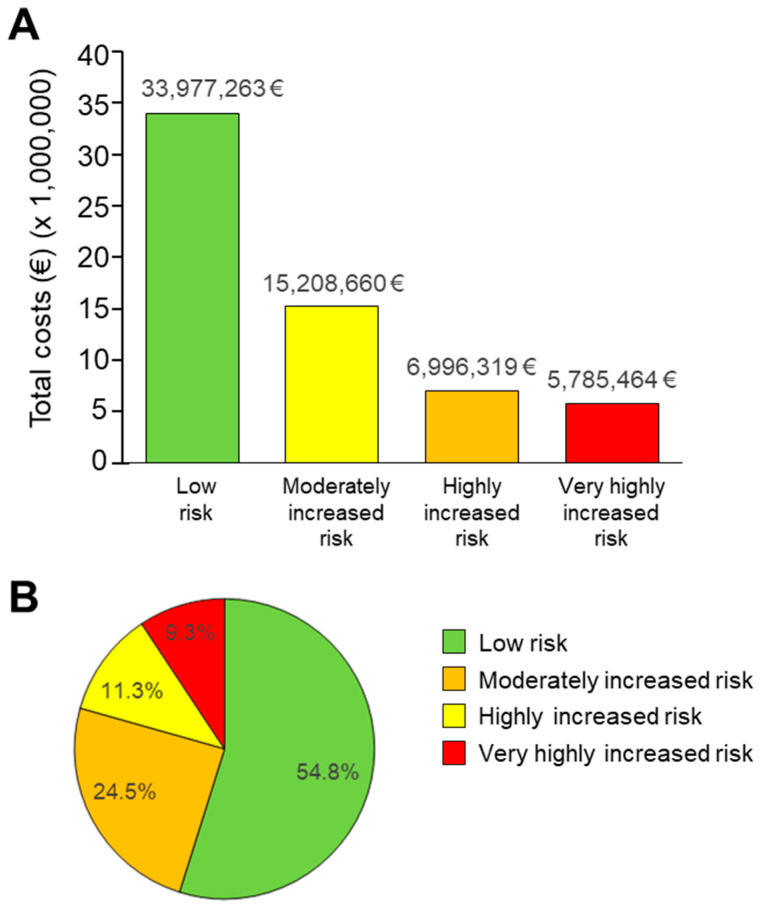
Total absolute costs associated to each KDIGO risk groups (**A**), and percent of costs of each KDIGO group (**B**).

The total costs (EUR) for each KDIGO category were highest in the low-risk group, inversely correlating with increasing risk (Figure 2). The patients at lower risk represented 54.8% of the total costs, those at moderately increased risk represented 24.5% of costs, those at highly increased risk represented 11.3% of costs, and those at very high risk represented 9.3% of costs. Taking into consideration the number of patients in each risk category, the mean cost per patient per year was EUR 3437 for low-risk patients, EUR 4936 for moderately increased risk patients, EUR 5899 for high-risk patients, and EUR 7389 for very high-risk patients. The total population costs per year were EUR 33,977,263, EUR 15,208,660, EUR 6,996,319, and EUR 5,785,465 for low, moderately increased, high and very high-risk patients, respectively.

The comparative statistical analysis provided statistically significant differences between all groups on the KDIGO scale for a *p*-value < 0.01.

**Figure 3:**
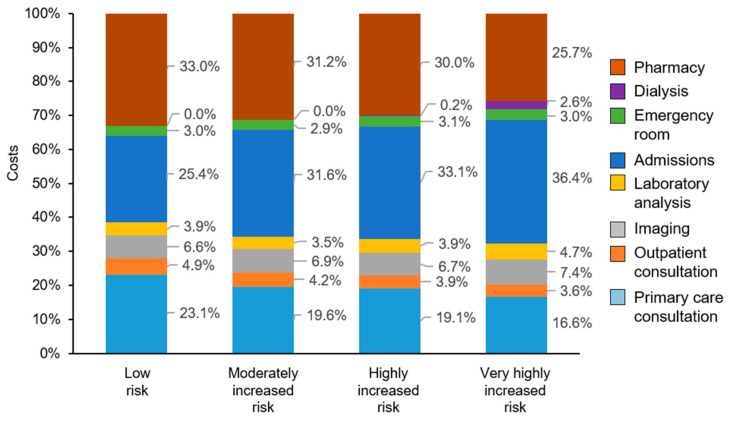
Distribution of total costs associated to each KDIGO risk group.

The distribution of costs was distinct for each risk category (Figure 3). In low-risk patients, the highest costs were pharmacy (33.0%), hospital admissions (25.4%), and primary care consultations (23.1%), while in patients at very high risk, the largest percentage of costs were hospital admissions (36.4%). Costs of dialysis, almost absent from other risk categories, represented 2.6% of the costs in very high-risk patients. The proportion of costs related with primary care consultations decreased from 23.1% in low-risk patients to 16.6% in very high-risk patients.

The total and mean costs per patient according to CRGs was highest in CRG 6 and CRG 8, respectively (Table 5). The highest total costs were those of patients in CRG 6 (EUR 34,941,906), while the mean costs were highest for patients in CRG 8 (EUR 12,141.0). The total costs of the 14,935 patients amounted to EUR 61,967,707.

A multiple linear regression analysis was carried out to determine main determinants of costs in relation to CRGs (Table 6). The results show that health states 7–9 and the severity levels 2–6 were determining factors (*p* < 0.01) of the increase in total cost. Health states 4–6, severity level 1, and eGFR were significant determinants of total cost reduction. 

## 4. Discussion

This study suggests that CKD in patients with T2D increases treatment costs and that the increment becomes evident from the early stages of CKD. The burden of care and budget impact that the increased consumption of resources and costs in early stages of CKD is critical due to the large number of patients affected. These results suggest that an early and integral approach to patients with T2D that improves control and limits the development of kidney disease would allow a substantial reduction in the burden of care and healthcare costs related to the management of these patients.

In this study, the observed prevalence of T2D (10.8%, 2015) was comparable to that of the overall population in Spain (13.8%, 2010) [3], and the mean age of the patients was also similar (67.8 versus 65.5 years). The higher prevalence in men than in women (up to 74 years of age) has also been observed at the national level [5], suggesting that the included cohort could be representative of the national population with T2D.

The study revealed that in this T2D population, only 14,935 out of 28,345 patients had eGFR and albuminuria determination during the study period. Out of those, 20% of the patients presented decreased glomerular filtration rate (eGFR < 60 mL/min/1.73 m^2^) and 21.3% of the patients presented moderately or severely increased albuminuria (>30 mg/g) resulting in an overall proportion of patients with increased CKD risk of 33.8%, according to the KDIGO classification. These values are comparable to those found in a study from another Mediterranean region, Catalonia (Spain), in which 22.9% and 19.5% of the patients with T2D presented renal impairment and albuminuria, respectively [8]. Albuminuria is a risk factor of myocardial infarction, stroke, total and cardiovascular death, and heart failure in patients with T2D compared to non-diabetes patients [34], although a considerable percentage of patients with T2D present reduced eGFR without increased albuminuria (3.4% in our study) [35]. High albuminuria and low eGFR are independent risk factors for cardiovascular morbidity, total mortality, and risk of CDK among patients with type 2 diabetes [36].

The US National Kidney Foundation defines CKD by the persistence of a low GFR and/or albuminuria during a period of at least three months [37]. Since our study was based on a single record of these parameters per patient, we can only assume that these patients were at risk of CKD at that point in time. The overall proportion of patients with CKD risk (33.8%) is in alignment with previous estimates of the prevalence of CKD in Spain ranging from 27.9 to 37.2% [7,8,9,10].

The results of the cost analysis indicate that, although patients in the low-risk category have the lowest mean overall costs per patient, they amount to more than half (54.8%) of the total costs of CKD among patients with T2D. Patients with low or moderately increased risk represent 87% of the population and account for about 80% of the total costs associated with the use of healthcare resources. In these low-risk patients, the highest expenditure is in medication (33.0%), mostly antidiabetic drugs. Our results also suggest that primary care is the main setting for most patients with T2D and CKD; although use of emergency services and hospitalization is the second cost driver from early stages, and strongly increases with increased risk. An earlier study showed that hospitalization due to nephropathy in patients with T2D in Spain represented 10.5% of all costs of complications of T2D [16].

In our study, it is evident that costs rise significantly already in low-risk and moderate-risk patients. Therefore, interventions designed to reduce the economic burden of CKD in T2D should be aimed to minimize progressive decline in kidney function starting with the earliest risk groups. Additionally, in our study, a sharp increase in costs was observed for patients presenting very poor renal function (stage G5, kidney failure), compared with patients in the G4 category, mostly due to dialysis. A similar pattern was observed in a recent study of the US population [28]. A study of patients in Singapore found that the cost increased with higher severity of CKD stage at baseline and was largely driven by hospitalization [29]. These studies suggest that interventions should also be designed to minimize progressive decline in kidney function in all patients at risk [26,27].

The analysis of the information from the electronic medical records has revealed patterns of multimorbidity associated with T2D [38,39,40,41,42]. In this study, we have used CRGs to classify patients according to their individual health state and risk, and assess multimorbidity. This classification could potentially be used in models to predict specific health states of patients and allow the development of models of resource utilization to improve resource allocation and to support clinicians’ decisions related to T2D patients. CRG-based patient categorization could also allow for more efficient and accurate comparison between related T2D comorbidities (e.g., cardiovascular disease).

This study presents some limitations which limit the conclusion reached. First, since there were missing data in the process of calculation of the total costs per patient, the calculated costs are approximate. In the case of laboratory and diagnostic imaging tests, it was unfeasible to obtain the cost of the entire catalog of all existing tests, generally due to coding or description problems. However, the included costs account for approximately 95% of the total costs, which is considered a good approximation of the real cost. Second, for costs of dialysis patients, it was not possible to obtain the details or the real cost of the dialysis sessions, so the averaged standard cost [43] has been applied to all patients in dialysis during 2015. Third, clinical data in this study (eGFR, albuminuria) derive from a single timepoint in the course of disease of the patients. Although this cross-sectional approach is indicative of risk of CKD, the true pattern of progression over time might not be reflected, as it could have changed with the administration of new pharmacological treatments. Fourth, the study did not assess indirect costs of CKD comorbidity in this population. The analysis of indirect costs should be the focus of further studies, as they could help identify cost drivers, thereby mitigating the high burden of disease on the patient.

According to the 2020 KDIGO guidelines [44] for the management of diabetes in CKD: Patients with diabetes and chronic kidney disease (CKD) should be treated with a comprehensive strategy to reduce risks of kidney disease progression and cardiovascular disease that includes the following strategies: appropriate nutrition (low salt and hypocaloric and low-fat dairy products, lower in processed meats, refined carbohydrates, and sweetened beverages), maintain physical activity, smoking cessation, optimization of metabolic control blood pressure and lipid management, RAS blockade, and using hypoglucemiants that have demonstrated cardio-renal benefit (SGLT2-i).

## 5. Conclusions

Our study shows that real-world data can provide additional evidence to drive care strategies in patients with T2D that can help delaying the progression of the disease and its complications, such as kidney failure. From the point of view of healthcare economics, these strategies should improve patient care while making T2D management more affordable for healthcare systems by reducing cost drivers. These strategies should include, for example, the use of treatments with proven renal benefit for T2D patients or the consistent monitoring of renal progression risk markers such as eGFR and albuminuria [45], which was shown to be done insufficiently in routine care in this study.

In conclusion, an accurate control of CKD risk factors for developing diabetes complications and the incorporation of personalized therapeutics from early stages may help achieving better patient outcomes and mitigate the economic burden of CKD in TD2 patients.

## Figures and Tables

**Table 1:** Characteristics and classification according to CRGs of patients with T2D and data on eGFR and albuminuria (*n* = 14,935) [31].

Main Health State (CRG)	*n* (%)	Age, Years, Mean (SD)	Gender, Male (%)	eGFR ^a^, Mean (SD)
1. Healthy	67 (0.5)	46.7 (14.8)	35.8	104.9 (19.7)
2. Significant acute disease	22 (0.2)	51.6 (14.0)	68.2	101.0 (19.4)
3. Single minor chronic disease	99 (0.7)	54.5 (14.4)	39.4	95.6 (20.0)
4. Minor chronic disease in multiple organ systems	97 (0.7)	59.4 (15.3)	32.0	92.3 (18.1)
5. Single moderate dominant or chronic disease	3.980 (26.7)	62.1 (13.9)	58.7	88.2 (18.1)
6. Significant chronic disease in multiple organ systems	8.438 (56.5)	70.0 (11.5)	49.8	77.0 (21.1)
7. Dominant chronic disease in three or more organ systems	1.759 (11.8)	75.1 (9.6)	54.4	67.0 (22.4)
8. Dominant neoplasms, metastases and complications	200 (1.3)	71.1 (9.9)	66.0	73.6 (24.8)
9. Severe diseases or extreme healthcare needs	55 (0.4)	62.8 (13.4)	60.0	75.6 (33.3)
Unclassified	218 (1.5)	77.1 (12.9)	54.4	56.3 (28.1)

^a^ Chronic Kidney Disease Epidemiology Collaboration (CKD-EPI). Abbreviations: CRG = Clinical Risk Group; eGFR = estimated glomerular filtration rate; SD = standard deviation.

**Table 2:** Distribution of the 14,935 patients according to eGFR and albuminuria categories according to the KDIGO 2012 CKD classification. Field coloring indicates risk of CKD: green, low risk; yellow: moderately increased risk; orange: high risk; red: very high risk [30].

				Albuminuria			
				A1	A2	A3	
				Normal to mildly increased	Moderately increased	Severely increased	
				<30 mg/g	30–300 mg/g	≥300 mg/g	Total
eGFR	G1	Normal or high	>90	4508 (30.2)	688 (4.6)	45 (0.3)	5241 (35.1)
	G2	Mildly decreased	60–89	5377 (36.0)	1210 (8.1)	113 (0.8)	6700 (44.9)
	G3a	Mildly to moderately decreased	45–59	1183 (7.9)	458 (3.1)	62 (0.4)	1703 (11.4)
	G3b	Moderately to severely decreased	30–44	570 (3.8)	323 (2.2)	75 (0.5)	968 (6.5)
	G4	Severely decreased	15–29	113 (0.8)	134 (0.9)	40 (0.3)	287 (1.9)
	G5	Kidney failure	<15	6 (0.04)	17 (0.1)	13 (0.1)	36 (0.2)
			Total	11,757 (78.7)	2830 (19.0)	348 (2.3)	14,935 (100)

Abbreviations: CKD = chronic kidney disease; eGFR = estimated glomerular filtration rate (mL/min per 1.73 m^2^); KDIGO = Kidney Disease: Improving Global Outcomes

**Table 3:** Use of healthcare resources in patients with T2D and CKD according to KDIGO categories [30,31].

Resource	KDIGO Category				Total
	Low	Moderately Increased	Highly Increased	Very Highly Increased	
**Use of resources (mean/patient)**					
Primary care consultations	17.45	21.11	25.00	27.28	19.32
Outpatient consultations	3.82	4.63	5.14	6.02	4.21
Hospital admissions	0.21	0.34	0.43	0.57	0.27
ER admissions	0.54	0.76	0.97	1.17	0.65
**Costs (EUR, mean/patient)**					
Outpatient consultations	170	206	228	268	187
Hospital admissions	875	1560	1950	2689	1197
Emergency room	102	144	184	221	124
Laboratory	135	173	228	347	162
Imaging	226	343	395	545	280
Pharmaceutical	1133	1540	1770	1895	1308

Abbreviations: CKD = chronic kidney disease; ER = emergency room; KDIGO = Kidney Disease: Improving Global Outcomes; T2D = type 2 diabetes.

**Table 4:** Mean (CI 95%) costs per patient associated to each KDIGO category (EUR).

				Albuminuria			
				A1	A2	A3	
				Normal to mildly increased	Moderately increased	Severely increased	
				<30 mg/g	30–300 mg/g	≥300 mg/g	Total
eGFR	G1	Normal or high	>90	3217 (3097–3336)	4816 (4323–5309)	6281 (3726–8,837)	3453 (3328–3577)
	G2	Mildly decreased	60–89	3622 (3511–3733)	5228 (4893–5562)	6302 (4888–7716)	3957 (3846–4069)
	G3a	Mildly to moderately decreased	45–59	4708 (4401–5015)	6000 (5424–6576)	6002 (4310–7695)	5103 (4830–5375)
	G3b	Moderately to severely decreased	30–44	5708 (5163–6253)	6354 (5697–7011)	6046 (4700–7391)	5949 (5547–6352)
	G4	Severely decreased	15–29	7859 (6420–9299)	8461 (7008–9913)	7623 (5271–9976)	8107 (7167–9048)
	G5	Kidney failure	<15	12,281 (9553–15,009)	14,899 (9220–20,578)	19,532 (10,675–28,389)	16,136 (11,933–20,338)
			Total	3722 (3640–3804)	5592 (5355–5830)	6837 (5980–7694)	4149 (4067–4231)
							
**Mean total costs per KDIGO category**			**3437**	**4936**	**5899**	**7389**	**4149**

**Table 5:** Total and mean costs (EUR) according to CRGs (*n* = 14,935).

CRG		Severity							Unclassified	Total
		0	1	2	3	4	5	6		
1. Healthy	*n*	10	3	26	-	5	23	-	-	67
	Total costs	3356	858	117,558	-	23,711	13,256	-	-	158,739
	Mean costs	335.6	286.0	4521.5	-	4742.3	576.3	-	-	2369.2
2. Significant acute disease	*n*	1	-	3	-	3	15	-	-	22
	Total costs	756	-	11,575	-	8005	20,784	-	-	41,120
	Mean costs	755.8	-	3858.2	-	2668.3	1385.6	-	-	1869.1
3. Single minor chronic disease	*n*	-	85	14	-	-	-	-	-	99
	Total costs	-	86,091	72,635	-	-	-	-	-	158,726
	Mean costs	-	1012.8	5188.2	-	-	-	-	-	1603.3
4. Minor chronic disease in multiple organ systems	*n*	-	38	33	25	1	-	-	-	97
	Total costs	-	64,459	54,951	113,189	1191	-	-	-	233,689
	Mean costs	-	1696.3	1662.1	4527.6	1191.0	-	-	-	2409.2
5. Single moderate dominant or chronic disease	*n*	-	2017	1,034	668	4	256	1	-	3980
	Total costs	-	3,346,092	2,594,747	1,731,109	16,455	873,560	6154	-	8,568,118
	Mean costs	-	1658.9	2509.4	2591.5	4113.6	3412.3	6154.2	-	2152.8
6. Significant chronic disease in multiple organ systems	*n*	-	3013	1887	1614	1236	637	51	-	8438
	Total costs	-	8,142,056	7,104,401	7,038,780	6,990,556	4,814,589	851,524	-	34,941,906
	Mean costs	-	2702.3	3764.9	4361.1	5655.8	7558.2	16,696.6	-	4141.0
7. Dominant chronic disease in three or more organ systems	*n*	-	437	269	738	191	86	38	-	1759
	Total costs	-	1,788,498	1,696,738	5,242,343	1,887,398	1,318,127	780,125	-	12,713,229
	Mean costs	-	4092.7	6307.6	7103.4	9881.7	15,327.1	20,529.6	-	7227.5
8. Dominant neoplasms. metastases and complications	*n*	-	1	39	78	67	15	-	-	200
	Total costs	-	2168	231,990	816,129	1,129,333	248,583	-	-	2,428,203
	Mean costs	-	2168.3	5948.5	10,463.2	16,855.7	16,572.2	-	-	12,141.0
9. Severe diseases or extreme healthcare needs	*n*	-	4	23	6	16	4	2	-	55
	Total costs	-	24,315	165,223	78,784	150,827	77,749	87,849	-	584,478
	Mean costs	-	6078.9	7183.6	13,130.7	9426.7	19,369.8	43,924.4	-	10,626.9
**Total**	***n***	**11**	**5598**	**3328**	**3129**	**1523**	**1036**	**92**	**218**	**14,935**
	**Total costs**	**4112**	**13,454,538**	**12,049,717**	**15,020,335**	**10,207,475**	**7,366,379**	**1,725,652**	**2,139,499**	**61,967,707**
	**Mean costs**	**373.8**	**2403.5**	**3620.7**	**4800.4**	**6702.2**	**7110.4**	**18,757.1**	**9814.2**	**4149.2**

**Table 6:** Multiple linear regression of cost determinants.

Variable	Regression Coefficient (SE)
Constant	5.881 (0.229) *
CRG 4. Minor chronic disease in multiple organ systems	0.466 (0.092) *
CRG 5. Single moderate dominant or chronic disease	0.433 (0.056) *
CRG 6. Significant chronic disease in multiple organ systems	0.896 (0.055) *
CRG 7. Dominant chronic disease in three or more organ systems	1.382 (0.058) *
CRG 8. Dominant neoplasms, metastases and complications	1.700 (0.075) *
CRG 9. Severe diseases or extreme healthcare needs	1.409 (0.113) *
Severity 1	0.845 (0.235) *
Severity 2	1.213 (0.235) *
Severity 3	1.333 (0.236) *
Severity 4	1.586 (0.236) *
Severity 5	1.730 (0.236) *
Severity 6	2.554 (0.247) *
eGFR G5	0.504 (0.135) *

Abbreviations: CRG = Clinical Risk Group; eGFR = estimated glomerular filtration rate; SE = standard error. * *p*-values < 0.01.

## Data Availability

The data presented in this study are available on request from the corresponding author.
